# The Stereoscopic Anisotropy Develops During Childhood

**DOI:** 10.1167/iovs.15-17766

**Published:** 2016-03-08

**Authors:** Ignacio Serrano-Pedraza, William Herbert, Laura Villa-Laso, Michael Widdall, Kathleen Vancleef, Jenny C. A. Read

**Affiliations:** 1 Facultad de Psicología Universidad Complutense de Madrid Campus de Somosaguas, Madrid, Spain; 2 Institute of Neuroscience, Henry Wellcome Building, Newcastle University, Framlington Place, Newcastle upon Tyne, United Kingdom; 3 Universidad Internacional de La Rioja, La Rioja, Spain

**Keywords:** stereoscopic anisotropy, stereoacuity, visual development

## Abstract

**Purpose:**

Human vision has a puzzling stereoscopic anisotropy: horizontal depth corrugations are easier to detect than vertical depth corrugations. To date, little is known about the function or the underlying mechanism responsible for this anisotropy. Here, we aim to find out whether this anisotropy is independent of age. To answer this, we compare detection thresholds for horizontal and vertical depth corrugations as a function of age.

**Methods:**

The depth corrugations were defined solely by the horizontal disparity of random dot patterns. The disparities depicted a horizontal or vertical sinusoidal depth corrugation of spatial frequency 0.1 cyc/deg. Detection thresholds were obtained using Bayesian adaptive staircases from a total of 159 subjects aged from 3 to 73 years. For each participant we computed the anisotropy index, defined as the log_10_-ratio of the detection threshold for vertical corrugations divided by that for horizontal.

**Results:**

Anisotropy index was highly variable between individuals but was positive in 87% of the participants. There was a significant correlation between anisotropy index and log-age (*r* = 0.21, *P* = 0.008) mainly driven by a significant difference between children and adults. In 67 children aged 3 to 13 years, the mean anisotropy index was 0.34 ± 0.38 (mean ± SD, meaning that vertical thresholds were on average 2.2 times the horizontal ones), compared with 0.59 ± 0.55 in 84 adults aged 18 to 73 years (vertical 3.9 times horizontal). This was mainly driven by a decline in the sensitivity to vertical corrugations. Children had poorer stereoacuity than adults, but had similar sensitivity to adults for horizontal corrugations and were actually more sensitive than adults to vertical corrugations.

**Conclusions:**

The fact that adults show stronger stereo anisotropy than children raises the possibility that visual experience plays a critical role in developing and strengthening the stereo anisotropy.

Binocular stereopsis is the process that allows us to recover the relative depth of objects from the binocular disparities between the images of the world projected into both eyes. Experiments using random dot stereograms^1^ to generate depth corrugations defined by horizontal disparities have shown the existence of a strong anisotropy in depth perception: at spatial frequencies lower than approximately 0.4 cyc/deg, horizontally oriented sinusoidal depth corrugations are much easier to detect than vertical corrugations.^2–6^ This anisotropy is similar to that found in sensitivity to disparity-defined slanted surfaces, where the sensitivity is greater for surfaces that rotated around the horizontal axis than for surfaces that rotated around the vertical axis.^7–11^

Despite the strong evidence for this stereoscopic anisotropy, little is known about its function or the underlying mechanisms involved. Recently, we proposed a novel mechanistic explanation for stereoscopic anisotropy: that humans have only a single spatial-frequency channel for vertically oriented disparity corrugations, tuned to frequencies above 0.4 cyc/deg, but have at least two channels for horizontal corrugations, with a channel tuned to frequencies below 0.4 cyc/deg responsible for the greater sensitivity observed for horizontal corrugations at low frequencies.^5^ However, direct tests of this speculation using masking experiments^4^ have found multiple disparity channels for both orientations. Witz and Hess,^12^ using spatially band-pass noise rather than random dots, also concluded that vertical and horizontal stereo corrugations are detected by multiple disparity channels.^13^ Thus, the single versus multiple disparity channel hypothesis for explaining the stereoscopic anisotropy cannot be sustained.

Another possible explanation of stereoscopic anisotropy relates to the anisotropy of summation fields. Tyler and Kontsevich^14^ found that summation fields extended only in the horizontal orientation and increased in size as the spatial frequency of the Gabor depth ripples was reduced (aspect ratio 4:1 cycles). They argue that the presence of high contrast vertical contours support stereopsis in vertical objects like branches, whereas for horizontal contours, only the surface texture supports stereoscopic depth. Thus, they suggest that horizontally elongated summation fields are needed to compensate the differences in disparity and luminance information present in natural images. According to this compensation mechanism, if we measure disparity thresholds for vertical and horizontal sinusoidal corrugations of low spatial frequency and only one cycle visible then, given that the size of the stimulus is smaller than the predicted summation field for that spatial frequency, we should expect to find higher disparity thresholds for horizontal than for vertical corrugations. Previous results have found exactly the opposite. Using a very low spatial frequency (0.05 cyc/deg) and only one cycle visible, the disparity thresholds for vertical corrugations were higher than for horizontal corrugations.^5^ Thus, this anisotropy of summation fields cannot explain the stereoscopic anisotropy.

Recently, van der Willigen et al.^6^ suggested that the origin of the stereoscopic anisotropy could be explained by a theoretical analysis of natural images. Having found the opposite orientation stereo anisotropy in barn owls, they explained this result suggesting that the brain could promote the use of disparity gradients, present in natural images, that are behaviorally most relevant. However, this explanation does not state whether the difference in sensitivity to horizontal/vertical disparity corrugations reflects a learnt response to the visual environment or whether it is present from birth.

One way to test this is to measure the stereoscopic anisotropy as a function of age. If this anisotropy changes with age then this would suggest that the anisotropy is not genetically “hardwired,” but develops as a result of exposure to the visual environment. In this research, we measured the stereoscopic anisotropy of 159 participants spanning a large age range (between 3 and 73 years). We compared disparity thresholds for horizontal and vertical corrugations using sinusoidal wave corrugations of low spatial frequency (see Fig. 2B). Results revealed that the stereoscopic anisotropy, although present in both children and adults, is stronger for adults than for children. This is the first developmental study of the stereoscopic anisotropy, and represents strong evidence that visual experience plays a critical role in developing and strengthening the orientation stereo anisotropy.

## Materials and Methods

We performed two psychophysical experiments, described in detail below. Experiment 1 measured the stereoacuity of the participants and Experiment 2 measured disparity thresholds for detecting vertical and horizontal sinusoidal corrugations.

### Subjects

A total of 161 participants (98 female) took part in the experiments. Seventy participants (35 female) were children aged from 3 to 13 years (see Table 1 for details); and 91 participants (63 female) were adults aged from 18 to 73 years. Adults were recruited through Newcastle University's (Framlington Place, Newcastle upon Tyne, United Kingdom) student population and a participant pool, while children were recruited at the Centre for Life science center (http://www.life.org.uk/). All participants (or their parents) reported having normal or corrected to normal visual acuity. All participants completed at least one of the two experiments in this study, but two participants failed to complete Experiment 1 and a different two failed to complete Experiment 2. Thus, we have data from 159 participants in each experiment.

**Table 1:**
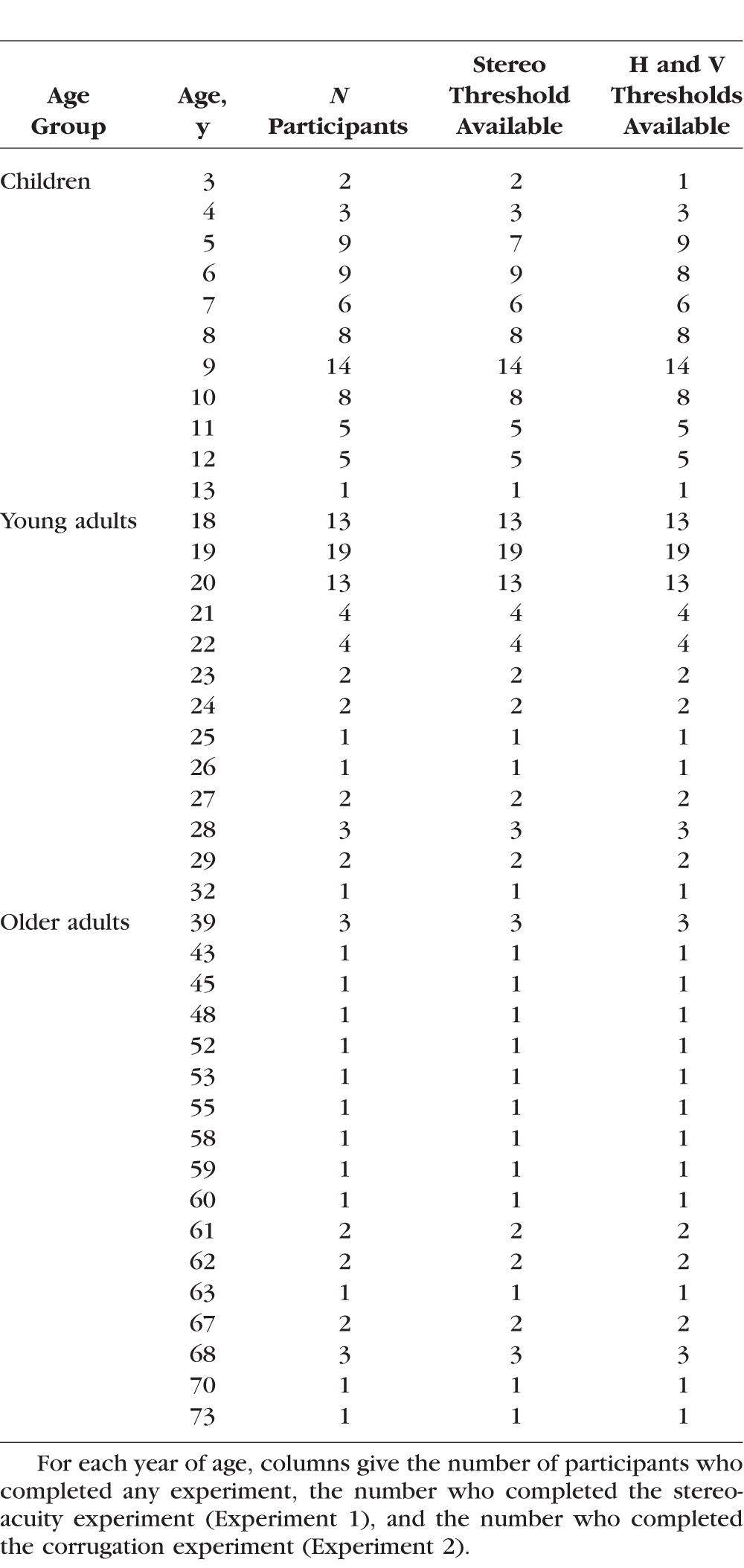
Age Distribution of Participants

**Figure 1:**
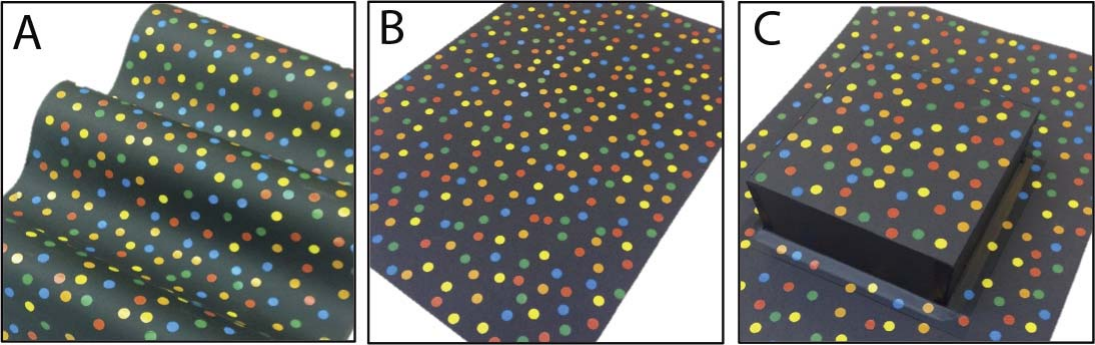
The mock stimulus used to aid children's understanding of the task: (**A**) sinusoidal depth corrugation, (**B**) zero disparity stimulus, (**C**) stereoacuity stimulus.

Adult participants were given an instruction sheet to read before completing the two experiments, while children were given oral explanations with the help of cardboard models depicting the depth structure of the stimuli (see Fig. 1). Adult participants, and parents or other accompanying adults of child participants, provided informed written consent. The study protocol was compliant with the Declaration of Helsinki and was approved by the Ethics Committee of the Newcastle University Faculty of Medical Sciences (approval number 00625).

**Figure 2:**
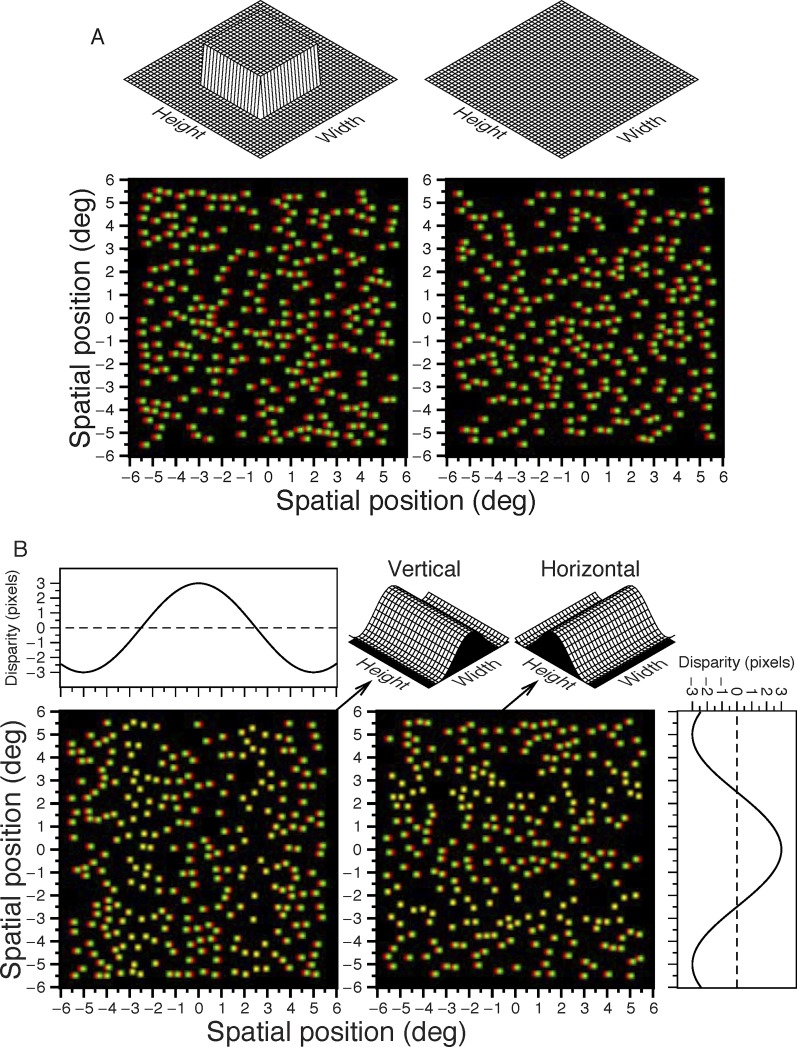
Anaglyph versions of the stimuli used in the experiments (for correct viewing, place the red filter in front of the left eye). In the real stimuli, dots were presented on a black background and there were no axis labels or numbers. (**A**) Stereoacuity experiment. The *left panel* contains the signal (disparate patch). The sketches above represent the expected 3D percept. (**B**) Horizontal/vertical corrugations. The *left anaglyph* corresponds to a vertical corrugation. The *right anaglyph* corresponds to a horizontal corrugation. The spatial frequency is 0.1 cyc/deg. The *panels* with the *lines* represent the disparities shown in the anaglyphs. The corresponding 3D percepts are shown in the *top right* part of the figure.

### Apparatus

Adult participants were tested in a dark room at Newcastle University and children performed the experiment on the same equipment in a dimmed area at the Newcastle Centre for Life, a public science center. The stimuli were presented on a 47-inch LG 3D monitor (47LD920; LG, Yeouido-dong, Seoul, South Korea) with a screen size of 104 cm × 58.5 cm. This is a patterned-retarder passive 3D monitor, where circular polarization is used to separate the left and right images. The spatial resolution of the monitor was 1920 × 1080 pixels and the refresh rate was 60 Hz. Observers sat at a viewing distance of 200 cm so a pixel subtended 54.65 seconds of arc (arcsec) on average. Adult participants used a forehead and chin rest. Children did not use a headrest, but head position was closely monitored by the experimenter. Observers wore appropriate passive 3D glasses (Sky 3D glasses, Middlesex, UK). Participants recorded their responses by pressing the left or right button of a standard computer mouse. The experiments were conducted using a DELL workstation (DELL, Round Rock, TX, USA), with a NVIDIA Quadro K600 graphics card (NVIDIA, Santa Clara, CA, USA), running Matlab (R2012b; MathWorks, Natick, MA, USA). The experiments were programmed using Psychophysics Toolbox extensions^15–17^ (in the public domain, www.psychtoolbox.org).

### Stimuli

The 3D was rendered with the monitor in standard 2D mode, using the line-interleaved stereo mode of Psychtoolbox's Psychimaging function. That is, our software generated left and right stimuli each 1920 pixels wide by 540 high, and interleaved them row by row to produce a single 1920 × 1080 image to send to the monitor.

The stimuli were dynamic random-dot stereograms consisting of bright dots on a black background. In order to make the stimulus more visually appealing for children, each dot was given a color generated by choosing the Red, Green, and Blue values independently from a uniform distribution between minimum and maximum luminance. Dots could overlap and occluded one another when they did so. The dots were drawn using Psychtoolbox's “Screen (‘DrawDots')” function, specifying circles 10 pixels in diameter with high-quality antialiasing. Because of the line interleaving, the dots appeared as ellipses on-screen, with a width of 10 pixels and a height of 20 physical pixels (9 × 18 arcmin). The dot density was such that if none of the dots had occluded each other, they would have occupied 16% of the stimulus; a stimulus filling the screen contained 2074 dots (density of 4.27 dots/deg^2^).

The disparity structure of the stimuli is described for each experiment below. On each trial, the disparity structure of the stimulus remained constant, but the dot pattern was updated (new random positions and colors) every frame (i.e., at 60 Hz). The stimulus was displayed until the subject made a response. No feedback was provided about correctness.

### Threshold Estimation

As in many aspects of perception, disparity thresholds are distributed roughly normally in log-space rather than linear. We accordingly work in log-disparity throughout; for example we report the geometric rather than the arithmetic mean of stereo thresholds in arcsec, which corresponds to the arithmetic mean of log thresholds.

To obtain participants' 75% threshold, we used a Bayesian staircase procedure, with some modifications designed to help child participants. For the likelihood function we used the logistic function of log-disparity (see details in the Supplementary information). The prior on the first trial was a uniform distribution between x_min_ = log_10_(0.5 arcsec) and x_max_ = log_10_(3600 arcsec).^18,19^ The posterior distribution on the threshold was updated after each trial by multiplying the prior by the likelihood function: *L* = Ψ(*x,θ*) for correct responses and *L* = (1-Ψ(*x,θ*)) for incorrect responses, where *x* is the log-disparity just presented. The posterior after *n* trials was taken to be the prior after (*n*-1) trials.

The stimulus disparity was set to be the mean of the currently-estimated posterior distribution, in the usual way.^20^ As the staircase proceeds, this becomes closer and closer to the true threshold, meaning that on most trials the target is not clearly visible to the participant, even if they are performing well above chance. We have found that naïve participants, especially children, can become demotivated as a result, and that it is helpful to include “easy trials” with a clearly visible stimulus.^21^ Accordingly, on each trial in this study, there was a 0.25 probability that the stimulus disparity would be chosen independently of the staircase and set to a value designed to be clearly visible. The result of these easy trials was still used to update the staircase. In most cases, the participant answered correctly and our estimate of the threshold barely changed. However, a wrong answer on an “easy trial” would cause our threshold estimate to increase, affecting the value chosen for the subsequent trial. The threshold estimate was taken to be the mean of the posterior distribution after 40 trials.^22^ The 68% confidence limits on this estimate were taken to be the 16% and 84% percentiles of this distribution and are used to draw error bars in the results figures.

### Experiment 1: Stereoacuity

Each participant first completed a measure of stereoacuity on a two-alternative forced choice (2AFC) disparity-detection task. Here, the random dot pattern covered the whole of the screen. The target was presented on one side of the screen (left or right). The target consisted of a square patch of dots 480 × 480 pixels (7.3° × 7.3°), that had a crossed disparity relative to the background dots (see Fig. 2A). The stimulus disparity was defined as the relative disparity between the square target and background. The target and background had equal and opposite disparity relative to the screen. This avoided monocular cues to target location if the viewer removed their glasses (if the background is in the screen plane and the target in front, the target appears blurred when viewed without glasses). Participants were asked to indicate which side (left or right) of the screen contained the target square.

To familiarize participants with the task, the first five trials had a large disparity (1800 arcsec) and a nonstereo color/luminance cue. These trials were not, of course, used to update the estimate of disparity threshold. On the first trial, the target dots were colored red, and the saturation of the red tinge was reduced over the next four trials. On the sixth trial, there was no color cue and the disparity was reduced to 42 arcsec, the mean of the initial prior distribution. Thereafter, the staircase proceeded as described above. If, however, the mean of the posterior distribution for threshold exceeded 400 arcsec, we began to mix in easy trials with a nonstereo cue (target dots colored red) to check the participant's understanding of the task. The probability that the next trial would contain a nonstereo cue was 50% when the mean of the posterior exceeded 1000 arcsec, 0% for less than 400 arcsec, and rose linearly from 0% to 50% as a function of mean-posterior from 400 to 1000 arcsec. We consider that threshold estimates greater than 1000 arcsec are not reliable, and we took them as indicating that the participant was effectively stereo blind.

Most participants performed 40 trials in total; three stopped earlier (after 27, 31, and 39 trials). Given the five initial nonstereo trials, this means that the threshold is estimated from a staircase procedure with at most 35 trials. This small number was necessary in order for young children to be willing to complete three such measurements (stereoacuity plus the two thresholds in Experiment 2). It is still large compared with clinical stereoacuity tests.

### Experiment 2: Detection Thresholds for Vertical and Horizontal Corrugations

Next, we measured participants' sensitivity for horizontally- and vertically-oriented depth corrugations defined by horizontal disparity (see Figs. 1A, 2B). The stimulus consisted of two square patches of random dots, each 760 × 760 pixels (12° × 12°), placed side by side on the screen. One patch had zero disparity, while the other depicted a sinusoidal depth corrugation with a frequency of 0.1 cyc/deg, a spatial frequency that has been found to produce a strong stereoscopic anisotropy.^2,5^ We used a detection task where the participant had to indicate whether the corrugation was in the left or right-hand patch, regardless of whether the corrugation was horizontal or vertical. Figure 2B shows an anaglyph version of this stimulus.

We measured the disparity amplitude, defined as half the relative disparity between peaks and troughs, required for performance at 75% correct. Since participants were by now familiar with the depth percept from the 3D monitor, we did not include any nonstereo cues in this experiment, although we continued to mix in 25% of “easy trials” as described above. Each threshold was estimated from 40 trials. Trials with horizontal and vertical corrugations were interleaved at random, so each participant completed 80 trials in this experiment (except for 3 participants who stopped early, after 60, 60, and 71 trials).

For each participant we computed an anisotropy index, defined as the log_10_-ratio of the detection thresholds for vertical versus horizontal corrugations.

## Results

### Stereoacuity: Distribution and Age-Dependence

**Figure 3:**
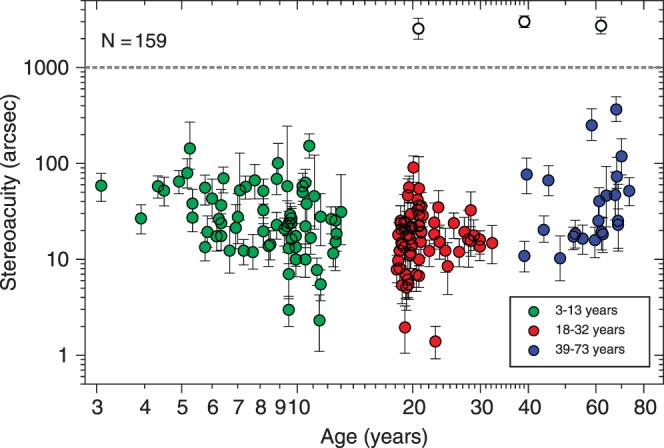
Results from Experiment 1. Stereoacuity thresholds (arcsec) as a function of age (years) for 159 subjects. *Error bars* represent the 68% confidence interval. The *upper dashed line* marks 1000 arcsec; thresholds above this are shown with *white symbols*. The limits of the vertical axis are the bounds of the posterior used in our staircase procedure (i.e., 0.5–3600 arcsec).

Figure 3 shows the results of Experiment 1. Figure 3 plots the stereoacuity thresholds as a function of age. Three of 159 subjects (2%) who performed the experiment had a threshold over 1000 arcsec (white dots), which is too poor to be measured accurately and should be considered “nil stereo,” so we did not consider these thresholds in the statistical analysis. The literature contains widely varying estimates of the prevalence of stereoblindness, but 2% is typical.^23,24^ Thresholds in the remaining 156 subjects ranged from 1.4 arcsec to 365 arcsec. Table 2 shows the means and the SDs for three different age groups: children (3–13 years), young adults (aged 18–32 years), and older adults (39–73 years).

**Table 2:**
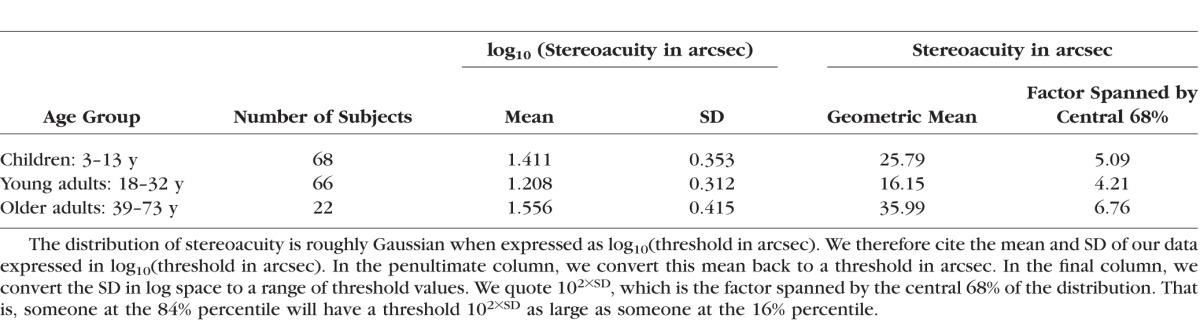
Means and SDs of the Stereoacuity Thresholds in Our Data Set, Divided by Age-Group, Excluding 3 Stereoblind Individuals (Thresholds > 1000 arcsec)

We see an improvement in measured stereoacuity between 3 years and 13 years (Pearson correlation coefficient on log_10_[thresholds] versus log_10_[age]: *r* = −0.348, *P* = 0.003, *N* = 68), then no change between 18 years and 32 years (*r* = −0.05, *P* = 0.67, *N* = 66), and a decline between 39 years and 73 years (*r* = 0.314, *P* = 0.15, *N* = 22). We performed a one-way ANOVA in order to compare the means (obtained from log_10_[thresholds]) of the three age-groups. We found a significant effect of group age on stereoacuity, (*F*_2,153_ = 10.49, *P* < 0.001). Post hoc analysis for multiple comparisons using the Bonferroni critical value showed a significant difference between children and young adults, and between young and older adults.

### Disparity Thresholds for Horizontal and Vertical Corrugations

**Figure 4:**
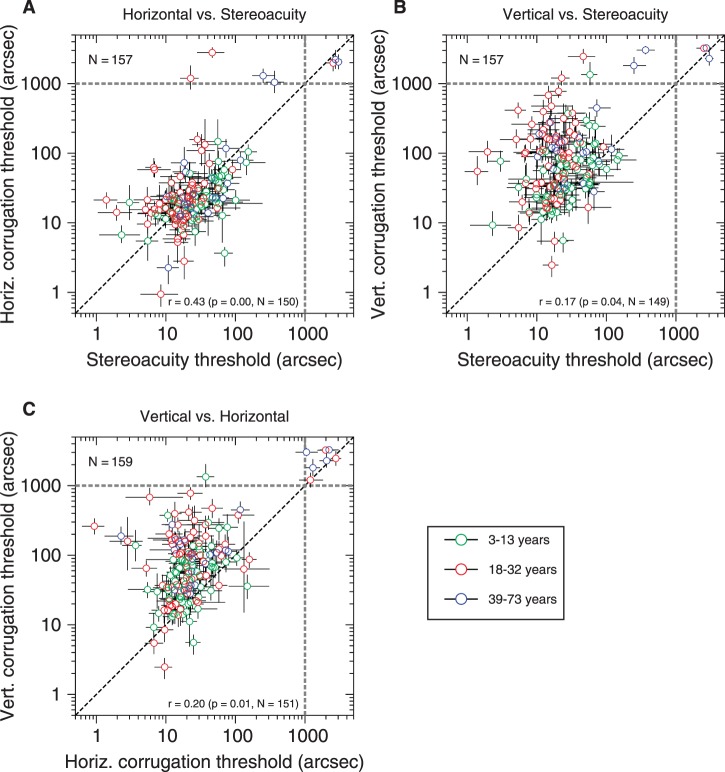
Disparity amplitude thresholds for detection of (**A**) horizontal and (**B**) vertical corrugations detection plotted against stereoacuity thresholds; (**C**) thresholds for vertical versus horizontal corrugations. *Diagonal dashed line* marks identity; *gray dashed lines* mark 1000 arcsec. *Bottom right* of each panel: Pearson correlation coefficient on the log-thresholds for thresholds less than 1000 arcsec. *Error bars* represent the 68% confidence interval.

Figures 4A and 4B plot the disparity amplitude threshold needed to detect a disparity corrugation (horizontal or vertical) against the stereoacuity threshold needed to detect a disparity-defined square. The gray-dashed lines mark 1000 arcsec. Points in the square region within these lines represent participants for whom we measured a threshold less than 1000 arcsec on both tests. Participants beyond these lines could not perform one or both tests reliably, and are therefore excluded from the following analysis. The *N* at the top left of each panel in Figure 4 gives the total number of participants for whom data were collected; the *N* at the bottom right gives the number after exclusion.

Figure 4A shows the results for horizontal corrugations. Horizontal corrugation thresholds and stereoacuity thresholds are moderately correlated (Pearson correlation coefficient on the log-thresholds: *r* = 0.43, *P* < 0.001, *N* = 150) as one would expect because both assess the quality of stereo vision. In fact, the two thresholds are not significantly different (*t*[149] = 0.59, *P* = 0.55, paired-sample *t*-test on log-thresholds). This is despite the fact that the stereoacuity threshold is defined as the relative disparity between the target and background, whereas the disparity amplitude threshold is half the relative disparity between the peaks and troughs of the waves, so one might have expected the disparity amplitude threshold to be half the stereoacuity threshold. Yet a *t*-test firmly rejects this hypothesis (*t*[149] = −9.57, *P* < 0.001, paired-sample *t*-test on log-threshold of horizontal corrugation amplitude threshold and log-half-threshold of stereoacuity).

Figure 4B shows the results for vertical corrugations. As expected given the stereo anisotropy, thresholds on the vertical corrugation detection task are much larger. The geometric mean threshold is 61.83 arcsec for vertical corrugations, compared with 20.58 arcsec for horizontal and 22.17 arcsec for stereoacuity. This difference is highly significant (paired-sample *t*-test comparing log-thresholds for vertical corrugations versus horizontal corrugations, *t*[150] = 11.77, *P* < 0.001, or versus stereoacuity, *t*[148] = 10.87, *P* < 0.001, including only thresholds <1000 arcsec).

Despite the higher thresholds on the vertical corrugation task, performance is slightly correlated both with thresholds on the horizontal corrugation task (Fig. 4C: *r* = 0.20, *P* = 0.01, *N* = 151) and with stereoacuity (Fig. 4B: *r* = 0.17, *P* = 0.04, *N* = 149).

### Stereo Anisotropy: Age-Dependence

**Figure 5:**
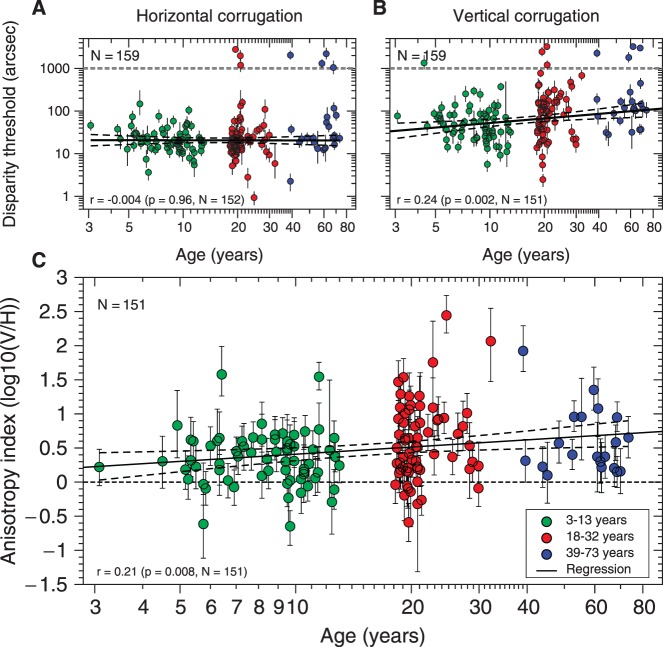
Results from Experiment 2. (**A**) Disparity thresholds for horizontal corrugation as a function of age. The fitted regression line for tresholds less than 1000 arcsec is H_disp._ = 10^(1.32-0.0046log10[age])^, (*dashed lines*: 95% regression confidence interval). (**B**) Disparity thresholds for vertical corrugation as a function of age. The fitted regression line thresholds less than 1000 arcsec is V_disp._ = 10^(1.364+0.354log10[age])^, (*dashed lines*: 95% regression confidence interval). *Top gray dashed lines* mark 1000 arcsec. *Bottom left of each panel*: Pearson correlation (log_10_(disparity) versus log_10_[age]) for tresholds less than 1000 arcsec. (**C**) Anisotropy index, AI = log_10_(V/H), as a function of age, for the 151 participants who could perform at both corrugation tasks with thresholds less than 1000 arcsec. The *fitted regression line* to all data is AI = 0.059 + 0.35 log_10_(age) (*dashed lines*: 95% regression confidence interval). *Dashed horizontal line* represents zero anisotropy. *Bottom left* of the panel shows Pearson correlation (AI versus log_10_[age]).

Figures 5A and 5B show the results for horizontal and vertical corrugations, respectively, as a function of age. Figure 5A shows that, remarkably, horizontal corrugation thresholds are not correlated with age (Pearson correlation coefficient on the log-thresholds: *r* = −0.004, *P* = 0.96, for thresholds < 1000 arcsec). We performed a one-way ANOVA to compare the horizontal thresholds (log_10_[thresholds]; thresholds < 1000 arcsec) for the three age groups specified in Table 2. We did not find significant differences (*F*_2,149_ = 0.74, *P* = 0.479; mean_Children_ = 1.324 [21.1 arcsec], SD_Children_ = 0.29, *N*_Children_ = 68; mean_Yadults_ = 1.28 [19.1 arcsec], SD_YAdults_ = 0.35, *N*_Yadults_ = 64; mean_OAdults_ = 1.379 [23.9 arcsec], SD_OAdults_ = 0.37, *N*_OAdults_ = 20). We also compared horizontal thresholds (log_10_[thresholds]; thresholds < 1000 arcsec) classifying the participants in two groups, children (≤ 13 years) and adults (≥ 18 years). Again, we did not find significant differences (*t*[150] = 0.36, *P* = 0.713, two-sample *t*-test; mean_children_ = 1.324 [21.1 arcsec], SD_children_ = 0.29, *N*_children_ = 68; mean_adults_ = 1.30 [19.95 arcsec], SD_adults_ = 0.36, *N*_adults_ = 84). Thus, interestingly, although children performed significantly worse than adults in the stereoacuity task (see Fig. 3), no difference was found for horizontal thresholds.

Figure 5B shows a mild but significant correlation between disparity thresholds for vertical corrugations and age (Pearson correlation coefficient on the log-thresholds: *r* = 0.243, *P* = 0.002, for thresholds < 1000 arcsec). We used ANOVA to compare vertical corrugation thresholds (log_10_[thresholds]; thresholds < 1000 arsec) for the three age groups (3–13 years, 18–32 years, and 39–73 years). We found significant differences (*F*_2,148_ = 5.829, *P* = 0.0036; mean_Children_ = 1.66 [45.80 arcsec], SD_Children_ = 0.37, *N*_Children_ = 67; mean_YAdults_ = 1.87 [74.4.1 arcsec], SD_YAdults_ = 0.51, *N*_YAdults_ = 64; mean_OAdults_ = 1.97 [93.47 arcsec], SD_OAdults_ = 0.32, *N*_OAdults_ = 20). Post hoc comparisons using the Bonferroni critical value showed significant differences between children (3–13 years) and young adults group (18–32 years) and between children (3–13 years) and older adults (39–73 years). We also compared vertical thresholds (log_10_[thresholds]; thresholds < 1000 arsec) classifying the participants in two groups, children (≤13 years) and adults (≥18 years). We found significant differences (*t*[149] = −3.29, *P* = 0.001, two-sample *t*-test; mean_children_ = 1.66 [45.80 arcsec], SD_children_ = 0.37, *N*_children_ = 67; mean_adults_ = 1.89 [78.55 arcsec], SD_adults_ = 0.476, *N*_adults_ = 84). Thus, for vertical corrugations, children performed better than adults. This is remarkable given that in the stereoacuity task children performed worse than adults.

To quantify the stereoscopic anisotropy, we define the anisotropy index to be the log_10_-ratio of the detection thresholds for vertical versus horizontal corrugations (see Figs. 5A, 5B). This is only meaningful for subjects who could perform both tasks, so the analysis reported in this section excludes 8 of 159 participants whose threshold on either corrugation task (see Fig. 4C), exceeded 1000 arcsec. The mean anisotropy index is 0.48 (SD = 0.5, *N* = 151). This means that on average, the detection threshold for vertical corrugations of 0.1 cyc/deg is a factor of 3 higher than for horizontal corrugations. This anisotropy index is highly significantly different from zero (*t*[150] = 11.76, *P* < 0.001, *t*-test). It is not significantly correlated with stereoacuity (*r =* −0.154, *P* = 0.06, Pearson's correlation coefficient, *N* = 149 nonstereoblind participants; *r* = −0.104, *P* = 0.20 if we include 3 stereoblind participants).

We used ANOVA to compare the anisotropy index for the three age groups (3–13 years, 18–32 years, and 39–73 years). We found significant differences (*F*_2,148_ = 4.9, *P* = 0.0087; mean_Children_ = 0.34, SD_Children_ = 0.38, *N*_Children_ = 67; mean_YAdults_ = 0.59, SD_YAdults_ = 0.57, *N*_YAdults_ = 64; mean_OAdults_ = 0.591, SD_OAdults_ = 0.477, *N*_OAdults_ = 20). Post hoc comparisons using the Bonferroni critical value showed a significant difference between children (3–13 years) and young adults group (18–32 years).

Figure 5C shows the anisotropy index as a function of age. If we consider all data together, there is a weak but significant increase with age (*r* = 0.21, *P* = 0.008, *N* = 151, for anisotropy versus log_10_[age]). The correlation is not driven by outliers such as the three high anisotropy indices visible between ages 20 and 40; if we remove the 10 points that are more than 2 SDs from the mean the correlation is unchanged (*r* = 0.19, *P* = 0.02, *N* = 141), and similarly if we remove the only 3-year old in our sample (*r* = 0.21, *P =* 0.009, *N* = 150). However, the correlation is driven mainly by a difference between children versus adults. There is no significant correlation between anisotropy and log-age within age-groups such as under 13 years or over 18 years. But if we compare children (≤13 years) versus adults (≥18 years), there is a highly significant difference. The mean anisotropy index in children is 0.34 (SD = 0.38, *N* = 67), corresponding to a threshold 2.2 times larger for vertical than for horizontal corrugations, and 0.59 (SD = 0.55, *N* = 84) in adults, corresponding to a threshold 3.9 times larger. These are both highly significantly different from zero (*t*[66]_children_ = 7.16, *t*[83]_adults_ = 9.8, *t*-test, *P <* 0.001 for both) and also significantly different from one another (*t*[149] = −3.14, *P* = 0.002, two-sample *t*-test).

**Figure 6:**
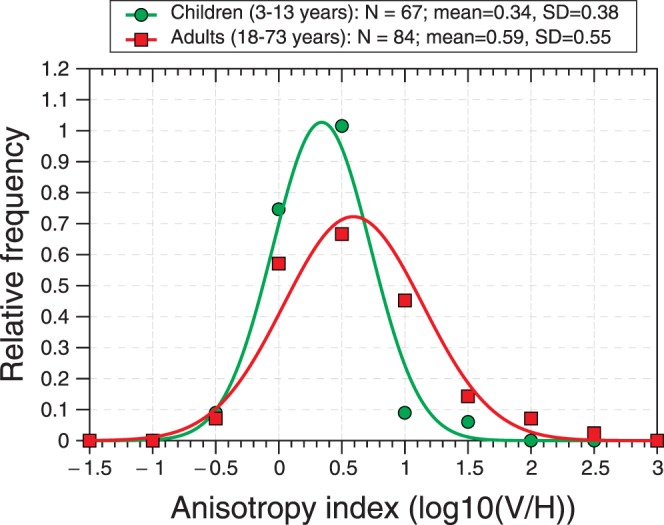
Distribution of stereo anisotropy index in children (*green circles*, *N* = 67) and adults (*red squares*, *N* = 84), from the data in Figure 5. *Symbols* show the frequency histogram; *curves* are Gaussians with the mean and SD of the anisotropy index for that age group.

Figure 6 shows the distribution of the stereo anisotropy index for children (green circles, ≤13-years old) and adults (red squares, ≥18-years old). It is worth pointing out that the intersubject variability was in fact slightly lower in children than in adults, suggesting that we were able to obtain reliable data from children and making it unlikely that their lower measured anisotropy index reflects greater measurement error.

## Discussion

Previous studies on the development of stereopsis have measured vergence, sensitivity to binocular correlation and, overwhelmingly, stereoacuity. Here, we assess the stereoscopic anisotropy (i.e., the greater sensitivity for horizontal depth corrugations [or to slant about a horizontal axis] than for vertical corrugations). To our knowledge, this is the first study to examine, across the lifespan, any property of stereopsis other than stereoacuity. We find that the orientation stereo anisotropy is present in childhood at the earliest ages we were able to examine (i.e., 3–5 years). However, it is more pronounced in adults (≥18 years) than in children (≤13 years). This difference implies that the stereo anisotropy increases during development.

Our confidence in this result is increased by the fact that the study reported here is our third study to find this result. Two pilot studies, using slightly different stimuli and methodology, each found the same basic result: a positive correlation between stereoscopic anisotropy and age, and a highly significant difference between the stereo anisotropy indices of children versus adults. Together, these studies contain data on 302 participants. Details of these studies are provided in the Supplementary Material.

### Detecting Corrugations

For horizontal corrugations, stereoacuity thresholds (i.e., the relative disparity between target and background) agreed with the disparity amplitude threshold (i.e., the relative disparity between peaks/troughs and zero disparity). Stereoacuity did not agree with the relative disparity between peaks and troughs of the corrugation. This suggests that, at least for the low frequency used here, corrugations are detected by comparing peaks and troughs to a zero-disparity reference (e.g., the other patch [Fig. 2B] or the sides of the monitor). However, this alone would not explain why the thresholds for vertical corrugations were so much higher, as described below, because the same strategy would have been available (using the top and bottom of the monitor as a reference, rather than its sides).

### Individual Differences in Stereo Anisotropy

Like other aspects of stereovision, stereo anisotropy is highly variable between individuals (Figs. 5, 6). This may explain why we could not detect a gradual increase in anisotropy throughout childhood or indeed adulthood. An additional problem is that we lack data between the ages of 13 and 18 years, reflecting the visitor demographics of the science center where the experiment was performed. The correlation between anisotropy index and age is thus driven mainly by the difference between preteen children and adults. Part of the reason for the wide variability in our study will certainly be the small number of trials on which each threshold was based: just 35. This was necessary in order to ensure that we could measure anisotropy in very young children, who are rarely willing to complete the hundreds of trials necessary for a really rigorous measure. However, other studies have also reported that the stereoscopic anisotropy shows wide variability between adult observers.^11^ Hibbard et al.^11^ computed thresholds as the mean estimates from three independent 3-up, 1-down staircases, compared with our single 35-trial staircase. By taking the logarithm of data in their Figure 3C, we deduce that the SD of the anisotropy index was 0.38 in their study, as compared with 0.55 in our 84 adults. At the extremes, 14% (6/42) of their young adult observers showed an anisotropy in the opposite direction (better for a vertical than a horizontal axis). (Hibbard et al. say in their text that “3 out of the 42 observers showed a small anisotropy in the opposite direction,” but they must mean “a significant anisotropy,” since it is clear from their data figures that in fact 6 observers have a measured ratio < 1. ) This agrees well with our results using a different stimulus and task (11% [9/84] of our adults and 16% [11/67] of our children showed opposite anisotropy). This confirms that the variability in our data is not simply due to measurement error. Rather, although the stereoscopic anisotropy is a robust phenomenon at the population level, individuals show considerable variation, implying that the developmental forces favoring anisotropy must be fairly weak. Intriguingly, individuals who are more sensitive to orientation differences than to spatial frequency differences in luminance are also more likely to show a strong stereoscopic anisotropy,^11^ presumably reflecting the fact that slant of a physical surface about a vertical axis introduces spatial frequency differences between the two eyes, whereas inclination about a horizontal axis introduces orientation differences.

It appears in Figures 4A and 4B that the interindividual variability in detection threshold may be larger for vertical corrugations than horizontal. This agrees with a previous study of slant thresholds^11^: observers showed greater variability in their ability to discriminate disparity-defined rotation of an originally frontoparallel plane about a vertical axis than about a horizontal axis.^11^ However, in our data the larger SD reflects the larger mean. If we consider coefficient of variation on log threshold (CV = SD[log-thresholds]/mean[log-thresholds]), there is little difference between the three tasks (CV = 0.25 for both vertical and horizontal corrugation detection, 0.27 for stereoacuity, considering only thresholds < 1000 arcsec. Including thresholds > 1000 arcsec increases the CV to about 0.36 for horizontal, 0.3 for vertical, and 0.33 for stereoacuity, but again reveals little difference between tasks.)

### Effect of Visual Acuity

Poor visual acuity, interocular acuity differences, and latent or manifest binocular misalignment can all affect stereopsis. Because we were already requiring young children to complete approximately 100 psychophysical trials, it was not feasible to include measurements of these quantities. We did measure stereoacuity, and excluded from analysis participants whose stereo threshold exceeded 1000 arcsec. We know that visual acuity improves up to the age of approximately 10, so if we had measured it, doubtless we would have found that our child participants had worse visual acuity than our young adults, as well as worse stereoacuity. Given the known links between visual acuity and stereoacuity,^25^ the age-related improvement in stereoacuity probably at least partly reflects the age-related improvement in visual acuity. However, it is hard to see how the age-related increase in the stereo anisotropy that we report could be a simple consequence of the age-related improvement in either visual acuity or stereoacuity. Lower acuity/stereoacuity would be expected to raise the detection thresholds for both grating orientations, but not to alter the ratio between them. In support of this, we find no evidence of a change in anisotropy index between young adults (18–32 years) versus older adults (39–73), despite the well-established decline in stereoacuity and visual acuity between these age-groups, confirming that a change in (stereo) acuity does not in itself alter anisotropy index. A similar argument applies to the concern that we did not formally screen participants for visual disorders such as amblyopia or uncorrected refractive error. Again, these problems would be expected to affect the visibility of both corrugations equally. Astigmatism would introduce differential blur in the horizontal and vertical directions, but our random-dot patterns are isotropic in luminance, and both grating types contain only horizontal disparities; they differ only in the distribution of horizontal disparities across the visual field. So, visual impairment would be expected to degrade perception of both corrugations equally, resulting in no change to the anisotropy index. It is therefore hard to see how our results could be an artefact due to the inclusion of participants with poor acuity or undetected binocular vision problems.

### The Reasons for the Stereoscopic Anisotropy

The reason for the stereoscopic anisotropy is still unclear. Other visual anisotropies have been related to the statistical distribution of the natural environment. For example, with luminance gratings, humans are more sensitive to orientations close to vertical or horizontal than to oblique orientations^26,27^ (the oblique effect). This effect could be related to the predominance of horizontal and vertical luminance edges in natural scenes.^28–30^ However, using broad-band oriented stimuli, the sensitivity was higher for oblique orientations and worst for horizontal orientations^30,31^ (the horizontal effect). Thus, in this case, it is not clear whether the visual system develops to match the most prevalent orientations in the visual world or whether the visual system perceptually discounts the most prevalent orientation in order to accommodate to the natural anisotropy.^30^ Regarding stereopsis, Sprague et al.^32^ recently showed that the horizontal and vertical disparities to which the visual system is most sensitive are those most commonly encountered in natural active viewing. To achieve this tuning, many aspects of the visual system are not genetically hard-wired, but depend critically on visual experience.^33,34^ The fact that the stereo anisotropy strengthens during development could also imply that it develops as an individual samples the natural environment. Our results show that although the sensitivity to horizontal corrugations does not change, or indeed slightly improves, with age, the sensitivity to vertical corrugations declines with age across the lifespan. Given that the stereoscopic anisotropy has been found mainly with low spatial frequency gratings (or slanted surfaces), an image-statistical account of the stereo anisotropy would require that in natural scenes, low-frequency disparity components are more common at horizontal orientations than at vertical (whereas high-frequency components are equally common at horizontal and vertical orientations). No one has yet produced evidence of such a difference.

It is also possible that the stereoscopic anisotropy may reflect differences in the functional significance, rather than frequency, of disparity gradients. For example, apparent rotation of a frontoparallel surface about a horizontal axis might occur because the viewer has swayed slightly forward or backward on their feet or because their head has tilted up or down on their neck (pitch). Detecting such a change could be important in maintaining postural stability. On the other hand, rotation about a vertical axis would occur if the person's head rotated on their neck (yaw). If unintended changes in pitch are more challenging to avoid than yaw, it might make sense to design the system to be more sensitive to visual cues indicating pitch than yaw.

### Stereoacuity Across the Lifespan

Although our sample is concentrated mainly on children and younger adults, our results are similar to previous reports about the development and decline of stereoacuity using standard clinical tests. We compared three different age-groups: “children” (3–13 years), “young adults” (18–32 years), and “older adults” (39–73 years). We found significant differences between young adults and children or older adults. Our results are consistent with previous studies, which reported an improvement in measured stereoacuity until approximately age 10 years,^21,35–41^ then no change until at least the age of 50 years, and thereafter a decline.^24,42–44^

Zaroff et al.^24^ plotted the frequency distribution of stereothresholds for 106 observers aged 15 to 59 years, measured on a front/back discrimination task with a stimulus duration of 100 ms and a viewing distance of 100 cm. Their distribution was a Gaussian function of log-disparity with a mean of 1.57 log_10_(arcsec) (corresponding to 37 arcsec) and SD of 0.227. Our “young adult” population has an average threshold of 16.15 arcsec, lower than the 37 arcsec of Zaroff et al.,^24^ probably reflecting our unlimited stimulus durations. The range in our population is larger than that of Zaroff et al.^24^ In our data-set, individuals whose stereoacuity is within ±1 SD of the mean can vary by a factor of nearly 9 in their thresholds, whereas this factor was only 3 in Zaroff et al.^24^ This does not reflect the different age-range because our results did not change appreciably if we included subjects from 15 to 59 years. It may reflect the more stringent selection criteria used in their study (all their observers had at least 20/30 Snellen acuity in each eye) or the larger number of trials performed by each subject. Given the differences in experimental procedure, the results are in fairly good agreement.

Despite the higher thresholds on the vertical corrugation task, we find that performance there is slightly correlated both with thresholds on the horizontal corrugation task (Fig. 4C) and with stereoacuity (Fig. 4B). This is the case even though the corrugations were presented at a low spatial frequency, 0.1 cyc/deg, below the peak of sensitivity at approximately 0.4 cyc/deg,^2,5,45^ whereas the stereoacuity task presented sharp edges containing high frequencies. This suggests that the same neuronal substrate ultimately limits all three tasks. A likely candidate would be the initial extraction of disparity in primary visual cortex.^46,47^

### The Stereoscopic Anisotropy Across the Lifespan

Stereopsis emerges in the first 11 and 18 weeks of life.^48^ Stereoacuity improves rapidly and reaches near adult levels at approximately 2 years of age, although improvement continues until around the age of 10,^49^ when visual maturation is generally considered to be complete^50^ (though recently, Giaschi et al.^51^ found that stereo thresholds in adults were significantly better than even 12- to 14-year olds^50^). At least part of the apparent improvement in stereoacuity between the ages of, say, 5 years and 10 years almost certainly reflects more general cognitive development, for example, greater willingness to engage with psychophysical testing, rather than a genuine improvement in perception.

Here, we show that the stereoscopic anisotropy continues to rise into adulthood, implying that stereopsis is still being refined and developed. This is unlikely to reflect cognitive development (e.g., greater willingness to engage or improved understanding of the task). Such cognitive factors should affect thresholds for horizontal and vertical corrugations equally, resulting in no change in anisotropy. Thus, this increasing anisotropy with age is likely to represent a genuine change in the perception of disparity gradients as the visual system matures. Intriguingly, this change is not simply an improvement; as we have seen, it appears to be at least partially mediated by a decline in the ability to perceive low-frequency vertical corrugations. Despite the fact that children have significantly poorer stereoacuity than adults and are no better at detecting horizontal depth corrugations, we found that children are significantly better than adults at detecting vertical depth corrugations. Unlike the decline in stereoacuity observed in over 50s, the decline in sensitivity to vertical depth corrugations is not a feature only of later life but appears to proceed more or less constantly throughout the lifespan (see Fig. 5B).

If the stereo anisotropy does reflect the statistics of the natural environment, this shift may reflect a continuing reallocation of neural resources fine-tuning the nervous system for its environment. Although the human visual system is capable of plasticity throughout the lifespan under special circumstances (e.g., as a response to pathology), it would be surprising if the visual system continues to fine-tune its allocation of resources so late in normal development.

To summarize one possible interpretation: sensitivity to disparity boundaries (stereoacuity) increases rapidly early in life, then more slowly, and is complete by the age of approximately 10 years. Sensitivity to disparity gradients emerges in parallel, initially with roughly isotropic sensitivity to disparity gradients along all axes. However, long after stereoacuity has stabilized, neural resources continue to be reallocated from detecting disparity gradients along horizontal axes to detecting disparity gradients along vertical axes.

## Conclusions

The stereo anisotropy emerges in early childhood but grows stronger with age. Although stereoacuity improves throughout early childhood and remains constant from visual maturation until old age, children are significantly better than adults at detecting vertical depth corrugations. The sensitivity to disparity-defined vertical depth corrugations declines from childhood, resulting in a gradual increase in stereo anisotropy. This shows that developmental changes in stereopsis are not simply a matter of improving sensitivity across the board. This alteration in the relative sensitivity to vertical and horizontal disparity gradients may reflect a gradual adaptation to features of the visual environment.

## Supplementary Material

Supplement 1
