# Supplementary material for: The Stereoscopic Anisotropy Develops During Childhood
Source: Invest Ophthalmol Vis Sci. 2016 Mar 8;57(3):960–70. doi: 10.1167/iovs.15-17766 (PMC4788095; doi:10.1167/iovs.15-17766)
Supplement: Supplement 1 [file i1552-5783-57-3-960-s01.pdf]

Supplementary information

The stereoscopic anisotropy develops during childhood

Ignacio Serrano-Pedraza, William Herbert, Laura Villa-Laso, Michael Widdall, Kathleen Vancleef, Jenny C. A. Read

In this document we present the data from two pilot experiments. Pilot study A was performed in the Faculty of Psychology of the Complutense University of Madrid, Spain, and was approved by the Ethics Committee of that university. In the pilot study B adult participants were tested at Newcastle University and children performed the experiment on the same equipment in a dimmed area at the Centre for Life.

Materials and Methods

Subjects

In both pilot studies we ran the Experiment 2 described in the main text. In pilot study A we tested 35 subjects (with ages between 4 and 66 years). In pilot B we tested 117 subjects (aged 5.5- 62.6 years)

Apparatus

In the main study and pilot study B, adult participants were tested at Newcastle University and children performed the experiment on the same equipment in a dimmed area at the Centre for Life.

In the pilot studies we used a 23-inch LG 3D monitor (D2342P) that also uses passive polarization (51 cm × 28.5 cm). The spatial resolution of both monitors was 1920 × 1080 pixels and the refresh rate was 60 Hz.

Observers sat at a viewing distance of 90 cm for both pilot studies so a pixel subtended 59.32 arcsec for pilot studies. Children indicated their responses by pressing large brightly colored buttons (AbleNet, Roseville, MN; www.ablenetinc.com).

The pilot study A was conducted using a Mac Pro, with ATI Radeon HD 2600 XT graphics card running Matlab (R2009b). The main study and pilot B was conducted using a DELL workstation, with a NVIDIA Quadro K600 graphics card, running Matlab (R2012b). All experiments were programmed using Psychophysics Toolbox extensions [1-3] (www.psychtoolbox.org).

Stimuli

The 3D was rendered with the monitor in standard 2D mode, using the line-interleaved stereo mode of Psychtoolbox's Psychimaging function. That is, our software generated left and right stimuli each 1920 pixels wide by 540 high, and interleaved them row by row to produce a single 1920 × 1080 image to send to the monitor. We used static random-dot patterns (i.e. the dot pattern did not update during a trial) consisting of white two-dimensional Gaussian dots with a standard deviation of 1 arc min with a density of 30 dots/deg^2 and without overlapping.

Threshold estimation

For all studies (pilots and main) we used Bayesian staircases. The model likelihood psychometric function was assumed to be a logistic function of log-disparity [4] :

$$\Psi(x;\theta) = g + \frac{1-\lambda-g}{1+\exp(-b[a+x-\theta])}$$

$$b = \frac{2}{\sigma}\ln\left(\frac{1-\lambda-g-\delta}{\delta}\right)$$

$$a = \frac{1}{b}\ln\left(\frac{1-\lambda-p}{p-g}\right)$$

where Ψ = the percentage of trials on which the participant answers correctly;

x = the logarithm to base 10 of disparity in arcsec;

p = the performance level defined as threshold in the main experiment 0.75 (0.85 in the pilot studies);

θ = the threshold in $\log_{10}$-disparity, i.e. the value of x at which performance reaches threshold;

λ = lapse rate, i.e. the false negative rate at maximum stimulus visibility, 0.05

g = guess rate, i.e. the probability which would be correct by chance, 0.5;

δ controls where we consider the top/bottom of the psychometric function, 0.01;

σ = spread parameter, controlling how rapidly performance increases, 1.

We measured the disparity amplitude, defined as half the relative disparity between peaks and troughs, required for performance at 85% correct. We used a two-interval forced choice task (2IFC) where each trial consisted of two presentations and the signal could randomly be presented in one of the two temporal intervals. Thus, the task was to indicate which interval (first or second) that contained the signal. Thresholds for horizontal and vertical corrugations were measured separately, in blocks. In the main study we included easy trials, as described in the paper. In the pilot studies no easy trials were used. Other details were as for the main study.

Comparison of main study with pilot studies

These conclusions of the main document agree with the pilot studies. Figure S1 repeats Figure 5C (main document), this time adding in data for an additional 152 participants from pilot studies A and B conducted respectively in Madrid (red line and red squares) and in Newcastle (green line and green triangles). The pilot studies also measured detection thresholds for horizontal and vertical disparity corrugations of 0.1 cycles per degree, and as before we include data only from participants who could perform at both corrugation tasks with thresholds < 1000 arcsec. As explained in the Methods section, the methods of the pilot studies differed in certain details from those of the main

study. For example, the random-dot patterns consisted of white static, non-overlapping Gaussian blobs rather than dynamic, overlapping hard-edged dots. Subjects were shown two patterns, one with zero disparity and one with the corrugation presented sequentially in a two-interval forced-choice design, each interval being displayed for 450 ms, and the staircases for horizontal/vertical corrugations were run in separate blocks rather than being interleaved. Figure S1 shows that despite these methodological differences, the results were very similar. We performed an analysis of covariance (ANCOVA) to compare the slopes of the three regression functions fitted to the three studies (Figure S1, blue, red, and green lines). The three slopes were not significantly different ($F_{2,296}$=0.57, P = 0.567). The black line of Figure S1 represents the regression for all data (N = 302). Once again, there was a significant correlation between anisotropy index and log-age, and once again this was mainly driven by a significant difference between adults and children, rather than a steady increase across the life-span.

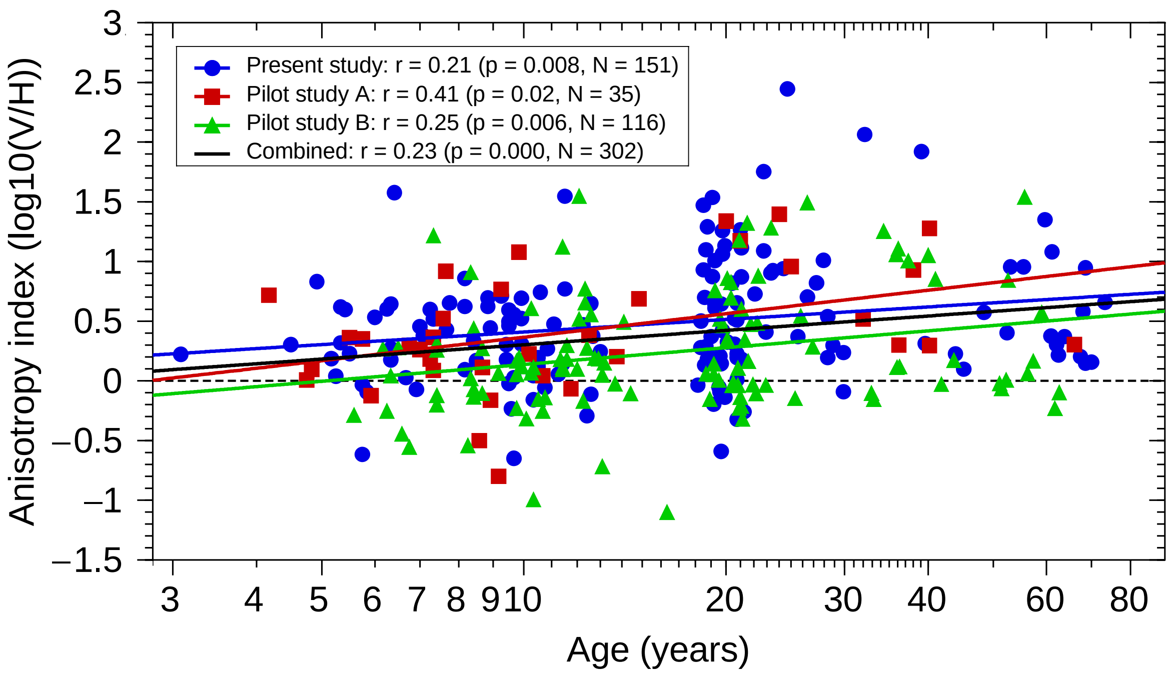

Figure S1. Anisotropy results for three studies: main study (blue circles), pilot study A (red squares), and pilot study B (green triangles). Symbols show the anisotropy index as a function of age. Thick lines show regression of anisotropy index on $\log_{10}$(age). The regression line for the main study (blue line) is AI_{main} = 0.059 + 0.35 $\log_{10}$(age). The regression line for the pilot study A (red line) is AI_{pilotA} = -0.288 + 0.653 $\log_{10}$(age). The regression line for the pilot study B (green line) is AI_{pilotB} = -0.33 + 0.467 $\log_{10}$(age). The regression line for the combined data (black line) is: $AI_{combined}$ = -0.097 + 0.399 $\log_{10}$(age). Dashed horizontal line represents zero anisotropy. The legend of the panel shows the Pearson correlation (AI vs. $\log_{10}$(age)) for each study.

Although we only show the anisotropy index for the pilot data, we also analysed the horizontal-vertical corrugation thresholds as we did with the results of the main experiment. We analysed both pilot studies together without the thresholds of the main experiment because in the pilot studies the performance level for the thresholds used in the Bayesian staircase was 85% (see Materials and Methods section, *threshold estimation*) and for the main experiment it was 75%. As a consequence, both thresholds were slightly higher in the pilot studies, although their ratio was unaffected.

For horizontal corrugation thresholds (thresholds < 1000 arcsec), we found no significant correlation for $\log_{10}$(thresholds) vs. $\log_{10}$(age) (r = -0.13, P = 0.094, N = 151). However, for vertical corrugation thresholds, we found a significant positive correlation (r = 0.16, P = 0.04, N = 151). Thus, despite the methodological differences, the main study successfully replicated the results of the previous pilot studies.

As in the main study, we compared horizontal and vertical thresholds ($\log_{10}$(thresholds); thresholds < 1000 arsec) for the two age-groups: children (<18 years) and adults (18+ years). For horizontal thresholds we found significant differences (P = 0.0157, two-tailed t-test; $\text{mean}_{\text{children}}$ = 1.49 (31.11 arcsec), SD_{children} = 0.39, N_{children} = 83; $\text{mean}_{\text{adults}}$ = 1.35 (22.49 arcsec), SD_{adults} = 0.28, N_{adults} = 68) and for vertical thresholds we also found significant differences (P = 0.044, two-tailed t-test; $\text{mean}_{\text{children}}$ = 1.64 (43.71 arcsec), SD_{children} = 0.43, N_{children} = 83; $\text{mean}_{\text{adults}}$= 1.79 (62.39 arcsec), SD_{adults} = 0.5, N_{adults} = 68). Thus, for vertical corrugations, the pilot studies and the main study consistently showed that children performed significantly better than adults. For horizontal corrugations, although the correlation between thresholds and age was not significant, in the pilot studies adults (18 + years) performed significantly better than children (< 18 years) whereas in the main study this difference was not significant.

Figure S2 shows the distribution of the stereo anisotropy index for children (green circles, <18 years) and adults (red squares, 18+ years). This is the same as Figure 6 in the paper, but includes data

from all 3 studies. The stereo anisotropy indices for both groups are highly significantly different from zero (P < 0.001 for both, t-test) and also highly significantly different from one another (P < 0.001, two-sample t-test). The mean anisotropy index was 0.23 (SD = 0.44) in children, corresponding to a threshold 1.7 times larger for vertical than for horizontal corrugations, and 0.52 (SD = 0.54) in adults, corresponding to a threshold 3.3 times larger.

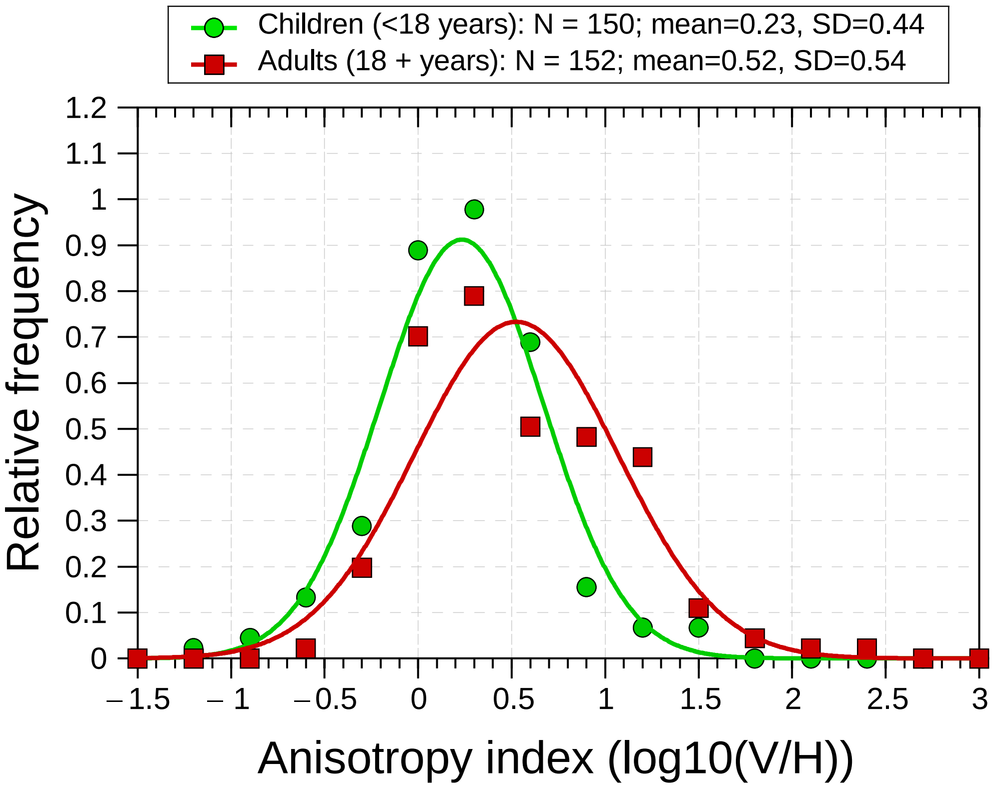

Figure S2. Distribution of stereo anisotropy index in children (green circles, N=150) and adults (red squares, N=152), from the pooled data in Figure S1. Symbols show the frequency histogram; curves are Gaussians with the mean and standard deviation of the anisotropy index for that age group.

References

1. Pelli DG. The VideoToolbox software for visual psychophysics: transforming numbers into movies. *Spat Vis* 1997;10:437-442.
2. Brainard DH. The Psychophysics Toolbox. *Spat Vis* 1997;10:433-436.
3. Kleiner M, Brainard D, Pelli D. What's new in Psychtoolbox-3? , *30th European Conference on Visual Perception.* Arezzo, Italy: Perception; 2007:14.
4. García-Pérez MA. Forced-choice staircases with fixed step sizes: asymptotic and small-sample properties. *Vision Res* 1998;38:1861-1881.
